# Exploration of the Effects of SGLT-2 Inhibitors and GLP-1 Receptor Agonists on Coronary Inflammation in Type 2 Diabetes Patients Based on the Peri-Coronary Fat Attenuation Index

**DOI:** 10.31083/RCM47415

**Published:** 2026-05-26

**Authors:** Tianxing Wang, Yanhong Li, Wenjing Yao, Sijin Zhang, Sihan Chi, Yongtao Wang, Chen Wang, Yi Dang, Feifei Zhang

**Affiliations:** ^1^Department of Graduate School, North China University of Science and Technology, 063210 Tangshan, Hebei, China; ^2^Department of Cardiology Center, Hebei General Hospital, 050051 Shijiazhuang, Hebei, China; ^3^Department of Graduate School, Hebei Medical University, 050017 Shijiazhuang, Hebei, China; ^4^Department of Graduate School, Hebei North University, 075000 Zhangjiakou, Hebei, China

**Keywords:** peri-coronary fat attenuation index, coronary inflammation, type 2 diabetes mellitus, sodium-dependent glucose transporter 2 inhibitor, glucagon-like peptide-1 receptor agonist

## Abstract

**Background:**

The peri-coronary fat attenuation index (FAI) is a novel imaging biomarker of inflammation. This study aimed to investigate the association between combination therapy with sodium–glucose transporter 2 inhibitors (SGLT-2i) and glucagon-like peptide-1 receptor agonists (GLP-1RAs) and coronary artery inflammation, as assessed by the peri-coronary FAI, in patients with type 2 diabetes mellitus (T2DM).

**Methods:**

This retrospective analysis included 292 patients with T2DM who underwent coronary computed tomography angiography (CCTA) at Hebei General Hospital. Patients were divided into three groups: (1) non-SGLT-2i/GLP-1RA users (non-users, n = 125): Patients not receiving SGLT-2i or GLP-1RA therapy; (2) SGLT-2i/GLP-1RA monotherapy group (mono-tx, n = 124): Patients treated with either SGLT-2i or GLP-1RA alone; (3) SGLT-2i + GLP-1RA combination therapy group (combo-tx, n = 43): Patients receiving concurrent SGLT-2i and GLP-1RA therapy. Clinical parameters, laboratory biomarkers, and the peri-coronary FAI of patients were collected and comparatively analyzed among the three groups. Finally, multivariate linear regression models were constructed to elucidate the independent association between combined GLP-1RA and SGLT-2i therapy and the peri-coronary FAI.

**Results:**

One-way analysis of variance (ANOVA) revealed significant differences in the peri-coronary FAI among the three therapy groups. Specifically, compared with the non-user group, the combo-tx group had significantly lower peri-coronary FAI values in the left circumflex artery (LCX) and left anterior descending artery (LAD). Compared with the mono-tx group, the combo-tx group also had a significantly lower LCX FAI. Multivariate regression analysis further confirmed that combination therapy was independently associated with a lower FAI in the LAD, LCX, and right coronary artery (RCA). Subgroup analysis revealed a significant interaction by sex in the association between treatment regimen and LCX FAI.

**Conclusion:**

The combined use of SGLT-2 inhibitors and GLP-1RAs may be associated with a decrease in the peri-coronary FAI in patients with T2DM, suggesting a potential role in reducing coronary inflammation. Thus, this combination therapy might offer advantages over monotherapy.

## 1. Introduction

Diabetes has become one of the most prevalent and severe chronic diseases in modern society. The estimated prevalence of diabetes worldwide in the 20–79-year-old population reached 10.5% (536.6 million people) in 2021, and forecasts suggest a surge to 12.2% by 2045 [1]. Worldwide, 32.2% of type 2 diabetes mellitus (T2DM) patients have comorbid cardiovascular disease (CVD), which has become the top cause of death in this population, with coronary artery disease and stroke being the primary contributors [2, 3]. Long-term hyperglycemia-induced endothelial dysfunction, vascular inflammation, and oxidative stress are strongly linked to the initiation and progression of CVD in T2DM patients [4]. Inflammation plays a central role in coronary atherosclerosis by promoting arterial plaque formation and progression, significantly augmenting the risk of cardiovascular disease [5, 6]. This establishes inflammation as a modifiable cardiovascular risk factor and therapeutic target, underscoring the need to explore treatment strategies to reduce coronary inflammation and improve cardiovascular outcomes in patients with T2DM.

The attenuation value of peri-coronary adipose tissue (PCAT) visualized by coronary computed tomography angiography (CCTA) serves as a noninvasive biomarker for evaluating coronary artery inflammation. As specialized epicardial adipose tissue (EAT) surrounding coronary arteries, PCAT has dual functions in maintaining vascular structural integrity and metabolic regulation [7, 8]. PCAT secretes proinflammatory cytokines such as Interleukin-6 (IL-6) and Tumor necrosis factor-α (TNF-α), establishing bidirectional signaling interactions with the wall of the coronary artery. Its dysfunction accelerates endothelial injury and atherosclerosis, whereas inflamed vascular walls reciprocally induce morphological changes in adjacent PCAT, such as enhanced lipolysis, inhibited lipogenesis, reduced lipid droplet volume, and increased water content, manifested as elevated computed tomography (CT) attenuation values. The peri-coronary fat attenuation index (FAI), assessed by CCTA, is defined as the mean CT value (between –190 and –30 HU) of adipose tissue within a 1× radius of the adventitial diameter of blood vessels and is used to quantify the degree of coronary inflammation [9, 10, 11]. The FAI exhibits high specificity for detecting local inflammation during the subclinical phase of coronary artery disease, prior to the development of significant stenosis. It enables anatomical localization of inflammation and facilitates the identification of unstable plaques and high-risk vascular territories. Moreover, FAI has emerged as a valuable tool for assessing treatment effects on coronary inflammation [12, 13].

Sodium-glucose transporter 2 inhibitors (SGLT-2i) and glucagon-like peptide-1 receptor agonists (GLP-1RA) have attracted considerable attention of their cardiovascular benefits [14, 15, 16]. Mechanistically, SGLT2 inhibitors improve cardiovascular outcomes through both glucose-dependent pathways (e.g., promoting urinary glucose excretion) and independent mechanisms (e.g., reducing cardiac preload, reducing inflammation, and inhibiting vascular/myocardial fibrosis) [17, 18]. GLP-1 receptor agonists activate GLP-1 receptors to delay gastric emptying and suppress appetite, promoting weight loss. They also directly improve vascular endothelial function, reduce oxidative stress and inflammation, inhibit atherosclerotic plaque progression, and lower cardiovascular risk by reducing blood pressure and heart rate while enhancing left ventricular systolic function [19, 20]. Clinical evidence shows that SGLT-2i significantly reduce the risk of cardiovascular death, heart failure hospitalization, and composite renal endpoints in patients with heart failure with reduced ejection fraction and chronic kidney disease (CKD) [21, 22]. GLP-1 receptor agonists decrease the risk of major adverse cardiovascular events (MACEs) in T2DM patients with atherosclerotic cardiovascular disease (ASCVD) [23, 24]. However, the specific anti-inflammatory effects of these two drug classes on coronary arteries remain undefined.

Therefore, this study sought to investigate whether combination therapy with SGLT-2i and GLP-1RA can more effectively alleviate subclinical coronary inflammation as characterized by FAI in patients with T2DM without major cardiovascular events, thereby providing imaging-based evidence for early-stage anti-atherosclerotic intervention in this high-risk population.

## 2. Materials and Methods

### 2.1 Study Population

This study was approved by the Ethics Committee of Hebei General Hospital (No. 2025-LW-0144), with a waiver of informed consent granted in accordance with relevant regulatory guidelines. A total of 516 consecutive T2DM patients who underwent CCTA at Hebei General Hospital between January 2023 and June 2024 were retrospectively included. The eligibility criteria were as follows: (1) age 18–75 years, (2) body mass index (BMI) ≥24 kg/m^2^, and (3) a confirmed T2DM diagnosis according to established clinical guidelines.

**Fig. 1.**
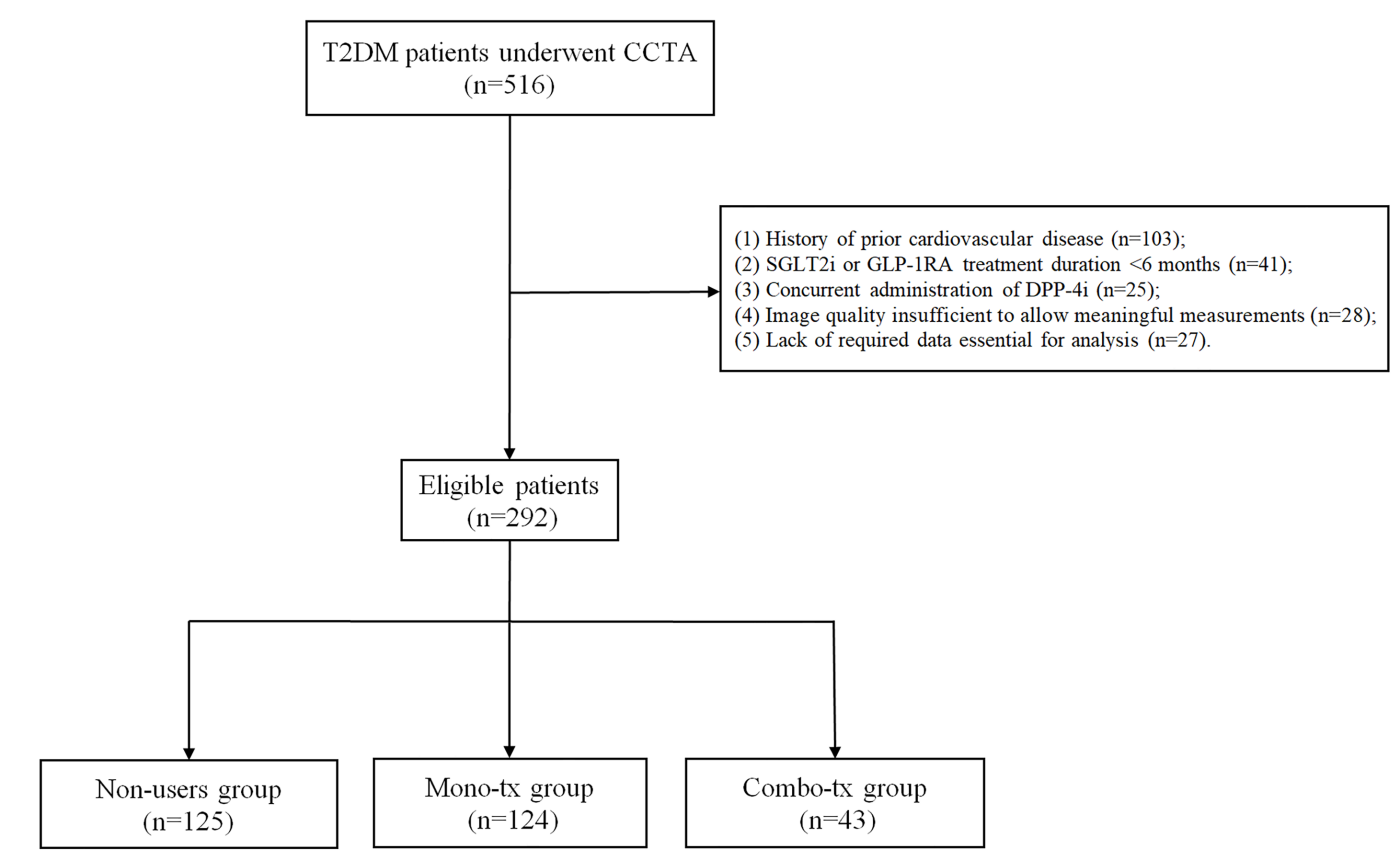
**Study flowchart**. T2DM, type 2 diabetes mellitus; CCTA, coronary computed tomography angiography; SGLT-2i, sodium-glucose transporter 2 inhibitors; GLP-1RA, glucagon-like peptide-1 receptor agonists; DPP-4i, dipeptidyl Peptidase-4 Inhibitors.

The exclusion criteria included: (1) prior cardiovascular diseases, including myocardial infarction, stroke, and heart failure, were included in the medical history; (2) SGLT-2i or GLP-1RA treatment duration <6 months; (3) concurrent administration of DPP-4 inhibitors (DPP-4i); (4) cases with image quality insufficient to allow meaningful measurements; and (5) lack of required data essential for analysis. Following application of the inclusion and exclusion criteria, 292 patients qualified for participation in the study (Fig. 1). Patients were divided into three groups according to their medication status: (1) Non-SGLT-2i/GLP-1RA users (Non-users, n = 125): Patients not receiving SGLT-2i or GLP-1RA therapy; (2) SGLT-2i/GLP-1RA monotherapy group (Mono-tx, n = 124): Patients treated with either SGLT-2i or GLP-1RA alone; (3) SGLT-2i+GLP-1RA combination therapy group (Combo-tx, n = 43): Patients receiving concurrent SGLT-2i and GLP-1RA therapy.

### 2.2 Medication Details

Specific details of the intervention drugs used by patients in this study are as follows: SGLT-2i included dapagliflozin and empagliflozin; GLP-1RA included semaglutide and liraglutide. Regarding dosages, the conventional daily dose of dapagliflozin and empagliflozin was 10 mg; semaglutide was administered at a conventional dose of 0.5 mg or 1.0 mg weekly, and liraglutide had a daily dose range of 0.6–1.8 mg. All dosing regimens aligned with standard clinical practice at our institution for achieving glycemic control and reducing cardiovascular risk. All patients met the enrollment criterion for medication duration (≥6 months), ensuring adequate exposure time for potential anti-inflammatory effects to manifest.

In the Mono-tx group (n = 124), 87 patients received SGLT-2i monotherapy, and 37 patients received GLP-1RA monotherapy. Within the combination therapy group (n = 43), the specific drug pairings included dapagliflozin or empagliflozin combined with either semaglutide or liraglutide, reflecting real-world clinical prescribing patterns. The initiation modes of medication in the combination therapy group (n = 43) were categorized into two types: 29 cases (67.4%) involved sequential addition of medications, and 14 cases (32.6%) involved simultaneous initiation of both drugs. The most common medication adjustment pathway involved patients initially receiving SGLT-2i therapy, followed by the addition of GLP-1RA due to suboptimal glycemic control or insufficient weight management. Another subset of patients began with GLP-1RA therapy, followed by SGLT-2i, with adjustments made based on a comprehensive assessment of glycemic, weight, cardiovascular, and renal benefits.

### 2.3 Baseline Data Collection

Clinical characteristics and laboratory parameters, including demographic data (age, sex, BMI), smoking history (current or past smoking ≥6 months), duration of T2DM, comorbidities (hypertension, hyperlipidemia), and medication profiles. Detailed hematological indices were systematically collected from the participants. The essential hematological parameters included fasting plasma glucose (FPG), glycated hemoglobin (hemoglobinA1c [HbA1c]), complete blood cell counts, and lipid panels.

### 2.4 CCTA Scan Acquisition

CCTA was performed at the Hebei General Hospital using a two-source CT scanner (Somatom ForceCT, Siemens Healthcare GmbH, Erlangen, Bavaria, Germany). Patients with a resting heart rate >70 beats per minute (bpm) received oral metoprolol tartrate before the examination to control their heart rate, with the goal of maintaining a resting heart rate at 60–70 bpm. The scanning range extended from the level of the tracheal carina and to 2 cm below the cardiac apex, covering the entire coronary artery tree from the origin to the distal branches. The scanning parameters were set according to individualization principles: the tube voltage was 80–120 kVp (100 kVp was preferred for patients with a body weight ≤70 kg, and 120 kVp was preferred for those with a body weight >70 kg), and the tube current-time product was 380–410 mAs. Electrocardiographic gating techniques were selected on the basis of heart rate: prospective ECG triggering was used for patients with a heart rate ≤65 bpm, whereas retrospective ECG gating was applied for those with a heart rate >65 bpm. Image reconstruction primarily uses the 75% phase of the R‒R interval (mid-diastole of the left ventricle).

### 2.5 Image Analysis and FAI Parameter Calculation

**Fig. 2.**
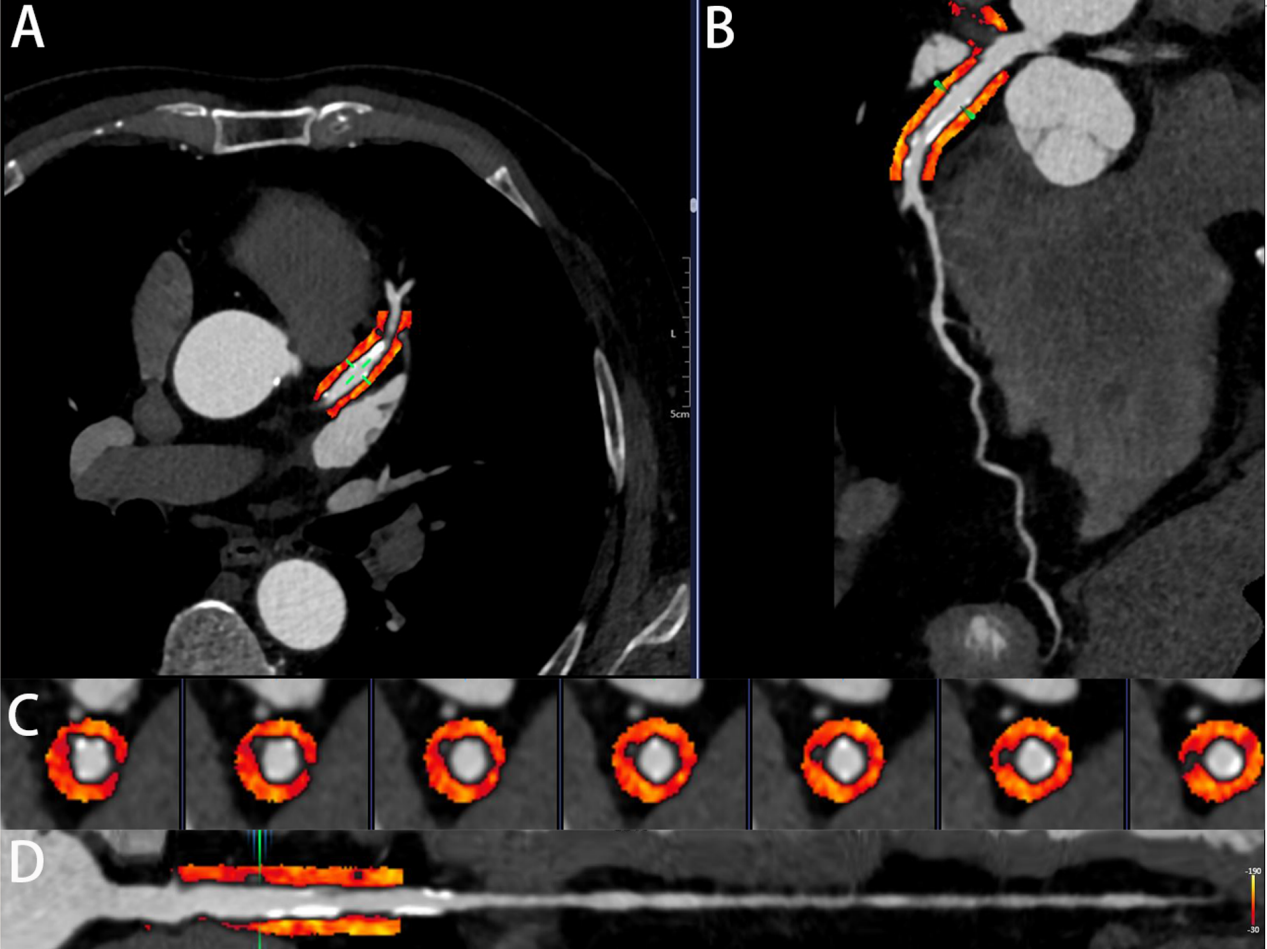
**An illustrative case of PCAT attenuation in the LAD**. (A) Cross-sectional view of PCAT. (B) Surface-recombined image of PCAT. (C) Straightened view of the proximal LAD segment. (D) Straightened view focusing on the proximal segment of the LAD. PCAT, peri-coronary adipose tissue; LAD, left anterior descending artery.

After professional radiographers at our hospital selected the optimal image of coronary artery sequences, the images for each patient were uploaded to FAI measurement software (Shukun Technology, version 1.0.4, Beijing, China), which automatically identified the main trunks of the left anterior descending artery (LAD), left circumflex artery (LCX), and right coronary artery (RCA). The region of interest (ROI) was defined as follows: for the RCA, ranging from 10 mm distal to the origin to 50 mm (extending 40 mm) to avoid aortic interference; for the LAD and LCX, 40-mm segments extended distally from the coronary ostia. The FAI was automatically calculated by the software as the mean CT value (attenuation coefficient between –190 and –30 HU) of PCAT within an annular range where the radial distance from the vascular outer wall equals the average vessel diameter (Fig. 2). All image analysts underwent standardized protocol training. The measurements were performed by two independent analysts who were blinded to the patients’ clinical data, and discrepancies were resolved by consensus negotiation to ensure the consistency and reproducibility of the measurement parameters.

### 2.6 Statistical Analysis

Categorical variables were reported as n (%) and compared via the chi-square test. Continuous variables with a normal distribution were presented as the mean ± standard deviation (SD) and analyzed with one-way ANOVA, followed by least significant difference *t* tests (LSD-t) for pairwise comparisons when significant overall differences were observed (*p* < 0.05). Skewed variables are reported as median (IQR) and were compared via the Kruskal‒Wallis test. Univariate linear regression (Model 1) was used to screen for variables associated with the peri-coronary FAI, followed by forward stepwise regression (The complete results of the univariate regression analysis and subsequent stepwise regression analysis are available in the **Supplementary Material**). Multivariate linear regression (Model 2) included clinically relevant confounders (age, sex, BMI, hypertension status, smoking history, T2DM duration, low-density lipoprotein cholesterol [LDL-C] level, coronary artery calcification score, left ventricular ejection fraction [LVEF], HbA1c level, white blood cell count, lymphocyte count, metformin, and statin use). Results are reported as unstandardized regression coefficients (b) with 95% confidence interval (CI). Trend tests (*p* for trend) were used to assess associations between SGLT-2i/GLP-1RA therapy and FAI. Subgroup analyses were stratified by sex, age, smoking history, hyperlipidemia, T2DM duration, and smoking history, with heterogeneity tests for monotherapy and combination therapy. Two-tailed tests were used for all analyses, with statistical significance defined as *p* < 0.05, using SPSS Statistics v27.0, IBM Corporation, Armonk, NY, USA.

## 3. Results

### 3.1 Patient Characteristics

**Fig. 3.**
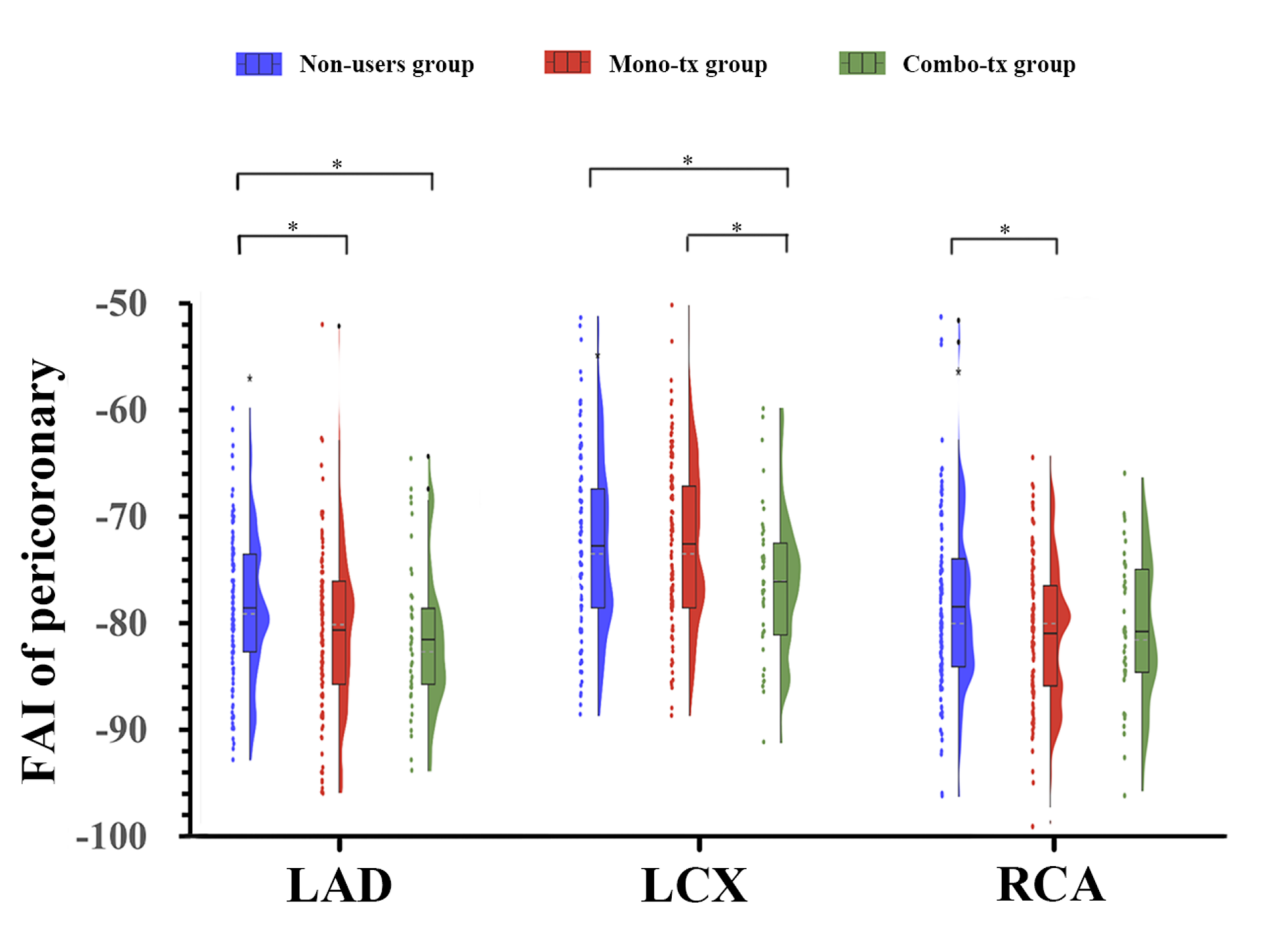
**Pairwise comparisons of peri-coronary FAI values among the three treatment groups**. *p < 0.05.

The clinical characteristics and baseline demographics of the patients are listed in Table 1. 292 patients were included in this retrospective analysis. Compared with those in the Mono-tx group and Non-users group, patients in the Combo-tx group were younger, with a mean age of 54.09 ± 10.78 years (*p* < 0.001), had a greater proportion of male patients (86.05% vs. 66.13%/59.20%, *p* = 0.006), had a greater proportion of metformin use (44.19% vs. 38.71%/26.40%, *p* = 0.041), and had higher hemoglobin levels (152.00 ± 13.41 vs. 138.55 ± 16.93/137.37 ± 18.99, *p* < 0.001). Among the three groups, no significant differences were noted in the duration of T2DM, cardiovascular risk factors (including smoking, hyperlipidemia, hypertension), use of hypoglycemic drugs (insulin, α-glucosidase inhibitors, sulfonylureas), blood pressure-lowering or lipid-modifying drugs, or HbA1c levels (*p* > 0.05). The levels of inflammatory markers and the coronary artery calcification score (CACS) also did not significantly differ among the three groups (*p* > 0.05). One-way ANOVA revealed significant intergroup differences in the peri-coronary FAI of the LAD, LCX, and RCA (*p* < 0.05). Further pairwise comparisons revealed that the FAI values of the LCX and LAD in the Combo-tx group were significantly decreased compared to the Non-users group, specifically the LAD: –81.86 ± 6.79 vs. –78.96 ± 6.67, mean difference (MD) = –2.90, *p* = 0.020; and the LCX: –76.57 ± 6.71 vs. –73.27 ± 8.11, MD = –3.30, *p* = 0.016. In addition, the LCX FAI values were significantly lower in the Combo-tx group compared with the Mono-tx group, i.e., LCX values (–76.57 ± 6.71 vs. –73.09 ± 7.65, MD = –3.48, 95% CI: –6.17 to 0.79, *p* = 0.011) (Fig. 3).

**Table 1.** **Baseline profiles of T2DM patients stratified by treatment regimens**.

		Total	Non-users group	Mono-tx group	Combo-tx group	*p*
		(n = 292)	(n = 125)	(n = 124)	(n = 43)	
Age (years)		59.65 ± 9.75	61.23 ± 9.25	59.99 ± 9.24	54.09 ± 10.78	<0.001
Male, n (%)		193 (66.10)	74 (59.20)	82 (66.13)	37 (86.05)	0.006
T2DM duration (years)		5.00 (1.25, 10.00)	4.00 (0.50, 10.00)	5.50 (2.00, 10.00)	7.00 (2.00, 13.00)	0.277
BMI (kg/m^2^)		27.68 (25.88, 29.74)	27.47 (25.71, 29.69)	27.77 (25.81, 29.75)	27.97 (26.39, 30.22)	0.416
Smoking		70 (23.97)	29 (23.20)	30 (24.19)	11 (25.58)	0.949
Hypertension, n (%)		209 (71.58)	95 (76.00)	88 (70.97)	26 (60.47)	0.147
hyperlipidemia, n (%)		153 (52.40)	60 (48.00)	68 (54.84)	25 (58.14)	0.400
Medication						
	Insulins (%)	80 (27.40)	30 (24.00)	37 (29.84)	13 (30.23)	0.530
	Metformin (%)	100 (34.25)	33 (26.40)	48 (38.71)	19 (44.19)	0.041
	α-glucosidase inhibitor (%)	133 (45.55)	52 (41.60)	66 (53.23)	15 (34.88)	0.058
	Sulfonylurea (%)	34 (11.64)	13 (10.40)	18 (14.52)	3 (6.98)	0.351
	SGLT-2i (%)	130 (44.52)	0 (0.00)	87 (70.16)	43 (100.00)	<0.001
	GLP-1RA (%)	80 (27.40)	0 (0.00)	37 (29.84)	43 (100.00)	<0.001
	Antihypertensive (%)	199 (68.15)	88 (70.40)	85 (68.55)	26 (60.47)	0.479
	Anti-platelet drugs (%)	237 (81.16)	105 (84.00)	98 (79.03)	34 (79.07)	0.563
	Statin (%)	259 (88.70)	110 (88.00)	109 (87.90)	40 (93.02)	0.625
	PCSK9i (%)	35 (11.99)	18 (14.40)	12 (9.68)	5 (11.63)	0.516
Laboratory indicators						
	Fast glucose (mmol/L)	7.48 (6.30, 9.82)	7.27 (6.10, 10.02)	7.79 (6.43, 9.72)	7.89 (6.83, 9.38)	0.899
	HbA1c (%)	7.30 (6.70, 8.40)	7.00 (6.49, 8.40)	7.43 (6.80, 8.45)	7.50 (7.05, 8.20)	0.586
	Total cholesterol (mmol/L)	4.426 ± 1.332	4.414 ± 1.371	4.373 ± 1.287	4.613 ± 1.361	0.593
	Triglycerides(mmol/L)	1.560 (1.080, 2.388)	1.490 (1.090, 2.290)	1.575 (1.055, 2.345)	1.890 (1.055, 3.030)	0.863
	HDL-C (mmol/L)	1.118 ± 0.257	1.124 ± 0.265	1.119 ± 0.261	1.099 ± 0.221	0.854
	LDL-C (mmol/L)	2.747 ± 0.932	2.734 ± 0.930	2.701 ± 0.918	2.918 ± 0.981	0.411
	Lipoprotein a (mg/L)	114.60 (49.95, 298.80)	112.50 (45.00, 273.03)	125.90 (57.32, 298.80)	92.70 (36.65, 332.10)	0.946
	Hemoglobin (g/L)	139.82 ± 17.92	137.37 ± 18.99	138.55 ± 16.93	150.56 ± 13.41	<0.001
	White blood cells (×10^9^/L)	6.69 ± 1.70	6.62 ± 1.71	6.67 ± 1.73	6.96 ± 1.62	0.525
	Neutrophils (×10^9^/L)	4.36 ± 1.51	4.36 ± 1.54	4.31 ± 1.52	4.54 ± 1.38	0.679
	Leukocytes (×10^9^/L)	1.80 ± 0.58	1.78 ± 0.61	1.81 ± 0.55	1.83 ± 0.58	0.863
	Platelet count (×10^9^/L)	222.76 ± 60.67	216.08 ± 62.03	227.86 ± 57.24	227.49 ± 65.67	0.266
	CRP (mg/L)	2.93 ± 1.83	2.91 ± 1.88	2.83 ± 1.81	3.24 ± 1.74	0.447
	LVEF (%)	63.74 ± 4.19	63.71 ± 4.05	63.54 ± 4.51	64.39 ± 3.61	0.514
CCTA parameters						
	CACS	52.36 (0.00, 273.94)	45.37 (0.00, 273.70)	44.12 (0.00, 227.24)	90.25 (0.00, 353.35)	0.574
FAI						
	LAD-PCAT (HU)	–80.25 ± 7.08	–78.96 ± 6.67	–81.00 ± 7.40	–81.86 ± 6.79	0.020
	LCX-PCAT (HU)	–73.68 ± 7.79	–73.27 ± 8.11	–73.09 ± 7.65	–76.57 ± 6.71	0.030
	RCA-PCAT (HU)	–80.32 ± 7.26	–78.94 ± 7.86	–81.40 ± 6.57	–81.23 ± 6.81	0.018

Continuous variables following a normal distribution were reported as the mean ± standard deviation, and skewed variables are reported as median (IQR). Categorical variables were presented as n (%). BMI, body mass index; HbA1c, hemoglobinA1c; PCSK9i, proprotein convertase subtilisin/kexin type 9 inhibitor; HDL-C, high-density lipoprotein cholesterol; LDL-C, low-density lipoprotein cholesterol; LVEF, left ventricular ejection fraction; CACS, coronary artery calcification score; LCX, left circumflex artery; RCA, right coronary artery; PCAT, peri-coronary adipose tissue; FAI, fat attenuation index.

### 3.2 Associations Between SGLT-2i/GLP-1RA Monotherapy or Combination Therapy and the Peri-Coronary FAI in T2DM Patients

Model 1 of the univariate linear regression indicated that monotherapy was correlated with a lower peri-coronary FAI of the LAD (b = –2.042, 95% CI: –3.783 to –0.301, *p* = 0.022) and RCA (b = –2.463, 95% CI: –4.249 to –0.677, *p* = 0.007) in T2DM patients, but was not significantly associated with a decreased peri-coronary FAI of the LCX (b = 0.179, 95% CI: –1.739 to 2.098, *p* > 0.05). Combination therapy was associated with a lower peri-coronary FAI of the LAD (b = –2.902, 95% CI: –5.331 to –0.474, *p* = 0.020) and LCX (b = –3.302, 95% CI: –5.978 to –0.625, *p* = 0.016), but it did not show a significant association with reduced peri-coronary FAI in the RCA (b = –2.293, 95% CI: –4.783 to 0.198, *p* = 0.072). After adjusting for relevant covariates (Model 2), monotherapy remained significantly associated with a decreased peri-coronary FAI of the LAD (b = –2.242, 95% CI: –3.997 to –0.448, *p* = 0.012) and RCA (b = –2.768, 95% CI: –4.521 to –1.015, *p* = 0.002), but not with the LCX (b = 0.018, 95% CI: –1.923 to 1.960, *p* = 0.985). Combination therapy was significantly associated with a decreased peri-coronary FAI in all three coronary arteries (LAD: b = –3.054, 95% CI: –5.625 to –0.484, *p* = 0.020; LCX: b = –3.602, 95% CI: –6.446 to –0.758, *p* = 0.013; RCA: b = –2.745, 95% CI: –5.313 to –0.177, *p* = 0.036). Trend tests (*p* for trend) in Model 2 revealed that combination therapy had a stronger effect on peri-coronary FAI reduction in the LAD (*p* = 0.005) and RCA (*p* = 0.005) than did monotherapy, with a significant linear trend (*p* ≤ 0.05). However, there was no significant linear trend in the effect on the LCX FAI between the two groups (*p* = 0.052), possibly because of the lack of significant efficacy of monotherapy on the LCX (b > 0, *p* > 0.05), leading to a discontinuous trend (Table 2).

**Table 2.** **Associations between three treatment groups and peri-coronary FAI: univariate and multivariate linear regression analyses**.

	Group	Model 1			Model 2		
		b (95% CI)	*p*	*p* for trend	b (95% CI)	*p*	*p* for trend
LAD-PCAT	Non-users	Reference		0.007	Reference		0.005
	Mono-tx	–2.042 (–3.783∼–0.301)	0.022		–2.242 (–3.997∼–0.448)	0.012	
	Combo-tx	–2.902 (–5.331∼–0.474)	0.020		–3.054 (–5.625∼–0.484)	0.020	
LCX-PCAT	Non-users	Reference		0.061	Reference		0.052
	Mono-tx	0.179 (–1.739∼2.098)	0.855		0.018 (–1.923∼1.960)	0.985	
	Combo-tx	–3.302 (–5.978∼–0.625)	0.016		–3.602 (–6.446∼–0.758)	0.013	
RCA-PCAT	Non-users	Reference		0.015	Reference		0.005
	Mono-tx	–2.463 (–4.249∼–0.677)	0.007		–2.768 (–4.521∼–1.015)	0.002	
	Combo-tx	–2.293 (–4.783∼0.198)	0.072		–2.745 (–5.313∼–0.177)	0.036	

Model 1: unadjusted; Model 2: adjusted for age, sex, BMI, hypertension status, T2DM duration, smoking history, statin, metformin, HbA1c, white blood cells, leukocytes, LDL-C, CACS, and LVEF. CI, confidence interval.

### 3.3 Subgroups Analysis

**Fig. 4.**
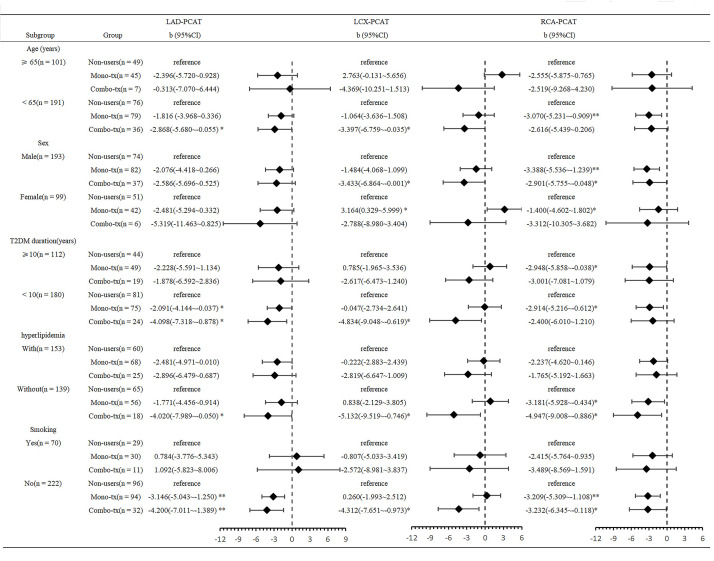
**Exploratory stratified analyses of associations between three treatment groups and peri-coronary FAI across different subgroups of T2DM patients**. *p < 0.05, **p < 0.01.

To investigate whether there were differences in the effects of SGLT-2i/GLP-1RA monotherapy or combination therapy on the peri-coronary FAI across different clinical subgroups, we conducted stratified analyses by sex, age, T2DM duration, hyperlipidemia status, and smoking history based on the multivariate linear regression model (Model 2). In addition, multivariate interaction tests between treatment regimens and subgroup variables were performed to assess the heterogeneity of treatment effects (Table 3). The interaction analysis revealed that only the interaction between treatment regimens and sex exerted a statistically significant effect on the FAI of LCX (*p*-interaction = 0.038). In contrast, no significant interactions were observed for other subgroup variables with respect to the FAI of the LAD, LCX, and RCA (all *p*-interaction > 0.05). Exploratory stratified analyses (Fig. 4) showed that in the sex subgroup, combination therapy was associated with a significant reduction in LCX FAI among male patients (b = –3.433, 95% CI: –6.864 to –0.001, *p* = 0.048). However, among female patients (n = 99, with only 6 receiving combination therapy), the point estimate for the association between monotherapy and LCX-PCAT was positive (b = 3.164, 95% CI: 0.329 to 5.999, *p* = 0.029), which contrasted with the overall downward trend. This discrepancy is most likely attributed to the limited sample size and random variation rather than a true biological effect. Although the interaction tests for other subgroups were not statistically significant, the point estimates from the exploratory stratified analyses suggested a numerical trend toward a stronger association between combination therapy and FAI reduction in patients aged <65 years, non-smokers, those without hyperlipidemia, or T2DM duration <10 years. This potential effect requires further validation in future large-sample prospective studies.

**Table 3.** **Interaction analysis between treatment regimens and subgroups on peri-coronary FAI**.

Interaction with treatment regimen	LAD-PCAT	LCX-PCAT	RCA-PCAT
	*p*-interaction	*p*-interaction	*p*-interaction
Sex (Male vs. Female)	0.798	0.038	0.526
Smoking (Smoker vs. Non-smoker)	0.138	0.668	0.690
Age (years)	0.433	0.522	0.794
Duration of T2DM (years)	0.780	0.882	0.483
Hyperlipidemia (Yes vs. No)	0.596	0.593	0.268

*p*-interaction: *p*-value for the interaction term (treatment regimens × subgroup variable) derived from multivariable linear regression models (based on Model 2).

## 4. Discussion

This study reports the results of the investigation of the relationship between the cardiovascular benefits of SGLT-2i combined with GLP-1RA and coronary inflammation in T2DM patients without known cardiovascular disease through the peri-coronary FAI, an imaging biomarker of coronary inflammation. This population is in a critical window period for atherosclerotic progression and urgently requires effective risk stratification and intervention strategies. As a tool capable of non-invasively detecting local coronary inflammation, FAI serves as an ideal surrogate endpoint for assessing early treatment responses. This study showed that SGLT-2i combined with GLP-1RA therapy was independently associated with a significant reduction in the FAI of the LAD, LCX, and RCA. After adjusting for confounders, the reduction in the LCX FAI with combination therapy was significantly greater than that with monotherapy, suggesting that combination therapy may confer cardiovascular benefits to T2DM patients by reducing coronary inflammation.

Multiple large-scale randomized controlled trials have confirmed that SGLT-2i and GLP-1RA can significantly reduce the risk of MACEs in T2DM patients [25, 26]. According to the 2025 American Diabetes Association (ADA) guidelines, in patients with T2DM complicated with ASCVD, heart failure, or CKD, SGLT-2i or GLP-1RA with proven cardiorenal protective evidence are preferentially recommended as first-line therapy (evidence level A) [27]. Observational studies have demonstrated the cardiovascular benefits of combined SGLT-2i and GLP-1RA therapy in patients with T2DM. For example, a UK CPRD cohort study showed that SGLT-2i/GLP-1RA combination therapy reduced the risk of MACEs by approximately 30% compared with monotherapy [28]. In a prospective observational study on T2DM patients with acute myocardial infarction (AMI), Marfella *et al*. [29] reported that, compared with either SGLT-2i or GLP-1RA monotherapy, combination therapy significantly reduced the risk of MACEs by 83%–84.6% and improved the myocardial salvage index (MSI). Further supporting these findings, a large retrospective cohort study by Chaiyakunapruk *et al*. [30] in the United States demonstrated that adding GLP-1RA to SGLT-2i reduced the risk of MACEs by 45%–46% in T2DM patients with ASCVD.

SGLT-2i reduce renal glucose reabsorption and promote glycosuria by inhibiting the activity of SGLT-2 protein in the proximal renal tubules. Their cardiorenal protective effects are achieved by alleviating volume overload to reduce cardiac workload, as well as by improving cellular energy metabolism. This process is often accompanied by activation of the AMP-activated protein kinase (*AMPK*) pathway, enhanced mitochondrial function, and mitigated oxidative stress. In addition, SGLT-2i exert prominent pleiotropic anti-inflammatory effects. Hyperglycemia and excessive volume overload under diabetic conditions lead to hyperactivation of the sympathetic nervous system, whereas SGLT-2i can lower sympathetic nerve tone through effective alleviation of volume overload, thereby suppressing neuroimmune-mediated inflammatory responses. SGLT-2i improve systemic metabolic disorders and insulin resistance via multiple mechanisms, including sustained glucose lowering, body weight reduction, and lipid profile regulation, thus exerting anti-inflammatory effects. GLP-1RA binds to GLP-1 receptors in the vascular wall, modulating endothelial cells, monocytes, macrophages, and vascular smooth muscle cells. This interaction not only enhances vascular endothelial function but also suppresses *NLRP3* inflammasome activation, thereby reducing the release of proinflammatory cytokines (e.g., IL-1β), decreasing monocyte adhesion molecule expression, and mitigating oxidative stress injury [31, 32]. The two drugs synergistically reduce oxidative stress and monocyte adhesion, which may jointly enhance the protective effect on the cardiovascular system.

The peri-coronary FAI, an emerging imaging biomarker based on CCTA, reflects coronary inflammation and plaque stability by quantifying attenuation value changes in PCAT. Studies have shown that the peri-coronary FAI can independently predict MACEs. Patients with high peri-coronary FAI values face a 3.29-fold greater risk of MACEs than those with low values [33, 34]. The CRISP-CT study demonstrated that increased FAI values in the RCA and LAD are significantly positively correlated with all-cause and cardiac mortality [35]. These findings suggest that inflammation-targeted therapies may improve patient prognosis. Notably, this study primarily included individuals with a BMI ≥24 kg/m^2^. Research has shown that overweight/obesity is strongly linked to the development and progression of T2DM, and that the mechanism involves inflammatory responses mediated by abnormal adipose tissue function. In the obese state, adipose tissue (including PCAT) secretes fewer protective factors with anti-inflammatory and antioxidant properties, such as adiponectin and omentin-1, and instead secretes large amounts of proinflammatory cytokines (IL-6 and TNF-α). These cytokines induce insulin resistance by interfering with insulin signaling pathways and can directly drive coronary inflammatory responses, leading to a functional shift in PCAT from “protective” to “harmful” [10, 36]. Therefore, excluding patients with a BMI <24 kg/m^2^ allowed for a more precise evaluation of the direct regulatory effect of these drugs on the “proinflammatory effect of adipose tissue in the obese state”, ensuring that the study results are more relevant to the target population requiring higher priority intervention in clinical practice.

A recent study confirmed that increased PCAT attenuation around the LAD in T2DM patients is significantly associated with cardiovascular events [37], suggesting that treatment strategies to reduce peri-coronary FAI may lower the risk of adverse cardiovascular events in T2DM patients. To date, investigations into the associations between SGLT-2i/GLP-1RA therapy and the peri-coronary FAI are relatively few. Studies by Liu *et al*. [38] and Li *et al*. [39] have shown that dapagliflozin and semaglutide may alleviate coronary inflammation in T2DM patients by lowering the peri-coronary FAI. Biesenbach *et al*. [40] demonstrated that liraglutide is linked to a lower LAD FAI in asymptomatic T2DM patients. However, the effect of the combined use of these two classes of drugs on the peri-coronary FAI remains unclear. Our study revealed that combined SGLT-2i and GLP-1RA treatment is negatively correlated with coronary inflammation in T2DM patients and has advantages over monotherapy in some cases. Subgroup analysis indicated that the association between combined SGLT-2i and GLP-1RA therapy and reduced FAI was consistent across patients stratified by age, T2DM duration, hyperlipidemia, or smoking history. A significant interaction was observed only for sex regarding its effect on LCX FAI, suggesting a potential sex-specific response. These findings offer insights for personalized treatment strategies in T2DM.

## 5. Limitations

This study has several limitations. First, due to the limited sample size, we grouped SGLT-2i and GLP-1RA into a single monotherapy group for analysis, thus failing to evaluate the independent effects of these two drug classes on the peri-coronary FAI, and were unable to fully exclude intra-group heterogeneity. Second, the retrospective study design is subject to selection bias (e.g., a higher proportion of young males in the combination therapy group and imbalances in medication use across some groups), and it was not possible to control for unmeasured confounding factors such as dietary patterns and physical activity. Although adjustments were made via multivariate models, the lack of randomization, blinding, and placebo control means this study can only reveal associations rather than causal relationships. Third, the relatively small overall sample size (n = 292) and the combination therapy subgroup (n = 43) may have limited statistical power, potentially affecting the results of some subgroup analyses. Fourth, we excluded patients with a BMI <24 kg/m^2^. While this focused the study on the high-risk overweight/obese population, it also restricted the generalization of the results to normal-weight patients with T2DM. Finally, the non-inclusion of indices such as epicardial fat volume and inflammatory cytokines limited the in-depth investigation of the potential anti-inflammatory mechanisms of combination therapy. Future multicenter, prospective randomized controlled trials (RCTs) are needed to validate the findings of this study, distinguish the independent effects of the two drugs, and expand the study population to enhance generalizability.

## 6. Conclusion

The combined use of SGLT-2 inhibitors and GLP-1 receptor agonists may be correlated with a decrease in the peri-coronary fat attenuation index in T2DM patients, suggesting their potential role in alleviating coronary inflammation. Compared with monotherapy, this combination therapy might offer potential advantages.

## Availability of Data and Materials

The datasets utilized or analyzed during the current study are available from the corresponding author upon reasonable request.
