# Supplementary material for: Exploration of the Effects of SGLT-2 Inhibitors and GLP-1 Receptor Agonists on Coronary Inflammation in Type 2 Diabetes Patients Based on the Peri-Coronary Fat Attenuation Index
Source: Rev Cardiovasc Med. 2026 May 26;27(5):47415. doi: 10.31083/RCM47415 (PMC13227378; doi:10.31083/RCM47415)
Supplement: Supplementary file 1 [file 2153-8174-27-5-47415-s1.zip › Supplementary Material.docx]

|  |  | LAD-PCAT |  | LCX-PCAT |  | RCA-PCAT |  |
| --- | --- | --- | --- | --- | --- | --- | --- |
| Subgroup | Group | b (95%CI) | P | b (95%CI) | P | b (95%CI) | P |
| Age (years) |  |  |  |  |  |  |  |
| ≥ 65(n = 101) | Non-users(n = 49) | reference |  | reference |  | reference |  |
|  | Mono-tx(n = 45) | -2.396(-5.720~0.928) | 0.155 | 2.763(-0.131~5.656) | 0.061 | -2.555(-5.875~0.765) | 0.130 |
|  | Combo-tx(n = 7) | -0.313(-7.070~6.444) | 0.927 | -4.369(-10.251~1.513) | 0.143 | -2.519(-9.268~4.230) | 0.460 |
| < 65(n = 191) | Non-users(n = 76) | reference |  | reference |  | reference |  |
|  | Mono-tx(n = 79) | -1.816 (-3.968~0.336) | 0.098 | -1.064(-3.636~1.508) | 0.415 | -3.070(-5.231~-0.909)** | 0.006 |
|  | Combo-tx(n = 36) | -2.868(-5.680~-0.055) * | 0.046 | -3.397(-6.759~-0.035)* | 0.048 | -2.616(-5.439~0.206) | 0.069 |
| Sex |  |  |  |  |  |  |  |
| Male(n = 193) | Non-users(n = 74) | reference |  | reference |  | reference |  |
|  | Mono-tx(n = 82) | -2.076(-4.418~0.266) | 0.082 | -1.484(-4.068~1.099) | 0.258 | -3.388(-5.536~-1.239)** | 0.002 |
|  | Combo-tx(n = 37) | -2.586(-5.696~0.525) | 0.103 | -3.433(-6.864~-0.001)* | 0.048 | -2.901(-5.755~-0.048)* | 0.046 |
| Female(n = 99) | Non-users(n = 51) | reference |  | reference |  | reference |  |
|  | Mono-tx(n = 42) | -2.481(-5.294~0.332) | 0.083 | 3.164(0.329~5.999) * | 0.029 | -1.400(-4.602~1.802)* | 0.387 |
|  | Combo-tx(n = 6) | -5.319(-11.463~0.825) | 0.089 | -2.788(-8.980~3.404) | 0.373 | -3.312(-10.305~3.682) | 0.349 |
| T2DM duration(years) |  |  |  |  |  |  |  |
| ≥10(n = 112) | Non-users(n = 44) | reference |  | reference |  | reference |  |
|  | Mono-tx(n = 49) | -2.228(-5.591~1.134) | 0.191 | 0.785(-1.965~3.536) | 0.572 | -2.948(-5.858~-0.038)* | 0.047 |
|  | Combo-tx(n = 19) | -1.878(-6.592~2.836) | 0.431 | -2.617(-6.473~1.240) | 0.181 | -3.001(-7.081~1.079) | 0.148 |
| < 10(n = 180) | Non-users(n = 81) | reference |  | reference |  | reference |  |
|  | Mono-tx(n = 75) | -2.091(-4.144~-0.037) * | 0.046 | -0.047(-2.734~2.641) | 0.973 | -2.914(-5.216~-0.612)* | 0.013 |
|  | Combo-tx(n = 24) | -4.098(-7.318~-0.878) * | 0.013 | -4.834(-9.048~-0.619)* | 0.025 | -2.400(-6.010~1.210) | 0.191 |
| hyperlipidemia |  |  |  |  |  |  |  |
| With(n = 153) | Non-users(n = 60) | reference |  | reference |  | reference |  |
|  | Mono-tx(n = 68) | -2.481(-4.971~0.010) | 0.051 | -0.222(-2.883~2.439) | 0.869 | -2.237(-4.620~0.146) | 0.066 |
|  | Combo-tx(n = 25) | -2.896(-6.479~0.687) | 0.112 | -2.819(-6.647~1.009) | 0.148 | -1.765(-5.192~1.663) | 0.310 |
| Without(n = 139) | Non-users(n = 65) | reference |  | reference |  | reference |  |
|  | Mono-tx(n = 56) | -1.771(-4.456~0.914) | 0.194 | 0.838(-2.129~3.805) | 0.577 | -3.181(-5.928~-0.434)* | 0.024 |
|  | Combo-tx(n = 18) | -4.020(-7.989~-0.050) * | 0.047 | -5.132(-9.519~-0.746)* | 0.022 | -4.947(-9.008~-0.886)* | 0.017 |
| Smoking |  |  |  |  |  |  |  |
| Yes(n = 70) | Non-users(n = 29) | reference |  | reference |  | reference |  |
|  | Mono-tx(n = 30) | 0.784(-3.776~5.343) | 0.732 | -0.807(-5.033~3.419) | 0.703 | -2.415(-5.764~0.935) | 0.154 |
|  | Combo-tx(n = 11) | 1.092(-5.823~8.006) | 0.753 | -2.572(-8.981~3.837) | 0.425 | -3.489(-8.569~1.591) | 0.174 |
| No(n = 222) | Non-users(n = 96) | reference |  | reference |  | reference |  |
|  | Mono-tx(n = 94) | -3.146(-5.043~-1.250) ** | 0.001 | 0.260(-1.993~2.512) | 0.820 | -3.209(-5.309~-1.108)** | 0.003 |
|  | Combo-tx(n = 32) | -4.200(-7.011~-1.389) ** | 0.004 | -4.312(-7.651~-0.973)* | 0.012 | -3.232(-6.345~-0.118)* | 0.042 |
